# Overview of lethal human coronaviruses

**DOI:** 10.1038/s41392-020-0190-2

**Published:** 2020-06-10

**Authors:** Bin Chen, Er-Kang Tian, Bin He, Lejin Tian, Ruiying Han, Shuangwen Wang, Qianrong Xiang, Shu Zhang, Toufic El Arnaout, Wei Cheng

**Affiliations:** 1 Division of Respiratory and Critical Care Medicine, State Key Laboratory of Biotherapy, West China Hospital of Sichuan University, Chengdu, 610041 China; 2 State Key Laboratory of Oral Diseases, National Clinical Research Center for Oral Diseases, West China Hospital of Stomatology, Sichuan University, Chengdu, 610041 China; 3 Department of Emergency Medicine, West China Hospital, Sichuan University, Chengdu, 610041 China; 4 Kappa Crystals Ltd., Dublin, Ireland

**Keywords:** Structural biology, Infectious diseases, Vaccines, Infection

## Abstract

Coronavirus infections of multiple origins have spread to date worldwide, causing severe respiratory diseases. Seven coronaviruses that infect humans have been identified: HCoV-229E, HCoV-OC43, HCoV-NL63, HCoV-HKU1, SARS-CoV, MERS-CoV, and SARS-CoV-2. Among them, SARS-CoV and MERS-CoV caused outbreaks in 2002 and 2012, respectively. SARS-CoV-2 (COVID-19) is the most recently discovered. It has created a severe worldwide outbreak beginning in late 2019, leading to date to over 4 million cases globally. Viruses are genetically simple, yet highly diverse. However, the recent outbreaks of SARS-CoV and MERS-CoV, and the ongoing outbreak of SARS-CoV-2, indicate that there remains a long way to go to identify and develop specific therapeutic treatments. Only after gaining a better understanding of their pathogenic mechanisms can we minimize viral pandemics. This paper mainly focuses on SARS-CoV, MERS-CoV, and SARS-CoV-2. Here, recent studies are summarized and reviewed, with a focus on virus–host interactions, vaccine-based and drug-targeted therapies, and the development of new approaches for clinical diagnosis and treatment.

## Introduction

Coronaviruses are crown-like particles with spikes protruding from their surface. They are enveloped viruses with single-stranded, positive-sense RNA (+ssRNA) genomes of approximately 26–32 kb, which is currently the largest known genome size for an RNA virus.^1^ RNA viruses are divided into five branches.^2,3^ Certain groups in a particular branch might be related to and/or share features with viruses classified in another branch. In Branch 1 are the leviviruses and eukaryotic relatives (e.g., mitoviruses, narnaviruses, ourmiaviruses); Branch 2 represents several +RNA virus families (of eukaryotes) (e.g., orders *Picornavirales* and *Nidovirales*, and the families *Caliciviridae*, *Potyviridae*, *Astroviridae*, and *Solemoviridae*) and several dsRNA viruses (e.g., partitiviruses and picobirnaviruses); In Branch 3 are certain +RNA viruses such as the supergroups of alphaviruses and offlaviviruses, the nodaviruses, tombusviruses, and many small and novel groups; In Branch 4 are dsRNA viruses such as the cystoviruses, reoviruses, and totiviruses; Branch 5 consists of −RNA viruses.^2^ Coronaviruses were classified as most likely related to Branch 2, and belong to the order *Nidovirales*, and the family *Coronaviridae*. In 2018, the International Committee on Taxonomy of Viruses divided the *Coronaviridae* into the *Orthocoronavirinae* and *Letovirinae* subfamilies.

**Fig. 1:**
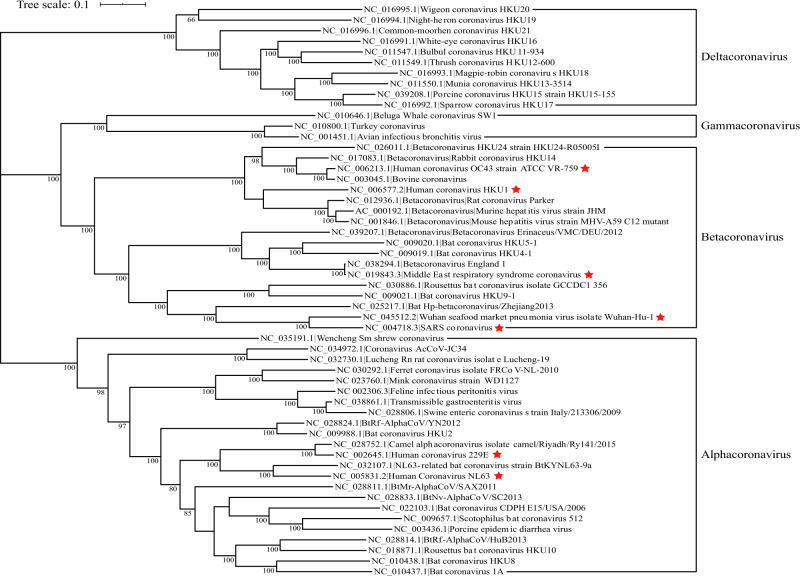
Phylogenetic tree of coronaviruses based on full-length genome sequences. All complete genome sequences of coronavirus were downloaded from the NCBI reference sequence database, RefSeq. The tree was constructed using maximum likelihood estimation (MLE) by MEGA X, with Clustal Omega as the multiple sequence alignment method, and 1000 bootstrap replicates. Only bootstraps ≥50% values are shown. The seven known human-infecting coronaviruses are indicated with a red star

According to their host targets, coronaviruses can also be divided into animal and human coronaviruses. Many diseases in domestic animals are related to animal coronaviruses, such as canine respiratory coronavirus, which causes respiratory disease in dogs.^4^ The highly pathogenic human coronaviruses belong to the subfamily *Coronavirinae* from the family *Coronaviridae*. The viruses in this subfamily group into four genera: *Alphacoronavirus*, *Betacoronavirus*, *Gammacoronavirus*, and *Deltacoronavirus*. The classic subgroup clusters are labeled 1a–1b for the alphacoronaviruses and 2a–2d for the betacoronaviruses. To date, including the recently discovered SARS-CoV-2, there are seven coronaviruses that infect humans. Human coronavirus (HCoV)-229E, HCoV-NL63, HCoV-OC43, or HCoV-HKU1 cause only the common cold,^5^ whereas the severe acute respiratory syndrome coronavirus (SARS-CoV) or the Middle East respiratory syndrome coronavirus (MERS-CoV) cause relatively high mortality and emerged in 2002^6^ and 2012,^7^ respectively. Notably, SARS-CoV-2 is currently causing a worldwide epidemic. SARS-CoV and MERS-CoV belong to subgroups 2b and 2c of the betacoronaviruses, respectively,^1,8^ whereas SARS-CoV-2 is a new member of the betacoronaviruses distinct from SARS-CoV and MERS-CoV.^9^ Figure 1 shows the phylogenetic tree of RNA viruses.

The estimated mutation rates of coronaviruses (CoVs) are moderate to high compared to those of other ssRNA viruses.^1^ There are two gene loci that are sites of variation in SARS-CoV. One of these sites is located in the spike (S) protein gene. The second major site of variation is the accessory gene open reading frame ORF8. In MERS-CoV, the major sites of variation are located in the S, ORF4b, and ORF3 genes.^2^ The major differences in the sequence of the S gene of SARS-CoV-2 are three short insertions in the N-terminal domain and changes in four out of five of the key residues in the receptor-binding motif.^9^ The organizations of the genome and gene expression are similar for all coronaviruses. ORF1a/b, located at the 5′ end, encodes 16 nonstructural proteins (NSPs) (named NSP1–NSP16); other ORFs at the 3′ end encode structural proteins, including the S, envelope (E), membrane (M), and nucleocapsid (N) proteins.

The mutations in SARS-CoV-2 have become a hot research topic. The surface proteins of SARS-CoV and BatCoV-RaTG13, the nearest potential bat precursors of SARS-CoV-2, have 76 and 97% sequence identity, respectively, to that of SARS-CoV-2.^10^ Compared to that of SARS-CoV, the antigenic surface of SARS-CoV-2 is highly divergent compared to other CoVs. Since its outbreak began at the end of 2019, SARS-CoV-2 has acquired mutations throughout its genome, and there are already hundreds of virus strains distributed worldwide. According to GISAID.org, as of May 8, there were 16,004 full genome sequences available, and the S clade of the full genome tree contains the largest group of viruses, indicating that mutations in ORF8-L84S are most frequent. The temporal resolution assumes an average nucleotide substitution rate of 5 × 10^−4^ substitutions per site per year. Based on these mutations, researchers found that SARS-CoV-2 can be divided into two subtypes. They reported that the SNPs at locations 8782 and 28,144 show strong linkage; the “CT” haplotype was defined as the “L” type because T28,144 encodes leucine, while the “TC” haplotype was defined as the “S” type because C28,144 encodes serine,^11^ which is in accordance with another study that divided SARS-CoV-2 into two types.^12^ The “L” type seems to be more prevalent and aggressive, but the “S” type may be ancestral, as sites 8782 and 28,144 were identical to the orthologous sites in the most closely related viruses.^13^ Patients may be infected by either or both types.^11^ From the GISAID update, compared with RaTG13, there are differences in the receptor-binding interface, which may have favored host switching. Data related to the sequence and mutations of SARS-CoV-2 are also available and continuously updated online through the China National Center for Bioinformation (CNCB) (https://bigd.big.ac.cn/ncov).

## Lethal human coronaviruses

### The history of SARS-CoV, MERS-CoV, and SARS-CoV-2

The first case of SARS was discovered in Foshan, China, in November 2002.^6^ Infections occurred through either direct or indirect contact with patients. By July 2003, SARS-CoV had spread to over 30 countries,^12^ causing 8096 reported cases and 774 deaths. After that, no additional infections were detected, and the SARS pandemic was declared over. SARS-CoV was first isolated from Himalayan palm civets (HPCs) from a live-animal market in Guangdong, China.^12,14^ Other animals, such as raccoon dogs (*Nyctereutes procyonoides*), along with human workers from the same market, also showed evidence of viral infection.^12^ Multiple studies have demonstrated that the reservoirs of several coronaviruses, including SARS-CoV-like and MERS-CoV-like viruses, were bats.^8,15^ Although palm civets might have been intermediary hosts of SARS-CoV,^16^ researchers often concluded there was no evidence that these animals were the ultimate sources of SARS-CoV, and the viruses cannot circulate directly in palm civets in the wild.^17^ In 2003, Guan and Zheng’s team investigated a live-animal retail market in Guangdong, focusing on recently captured wild animals and human consumption. The animals sampled included seven wild and one domestic animal species. They collected nasal and fecal samples with swabs and then used reverse transcription-polymerase chain reaction (RT-PCR) to test for viral nucleic acid from the N gene of the human SARS-CoV. The RT-PCR assay results showed that samples from four of six HPCs were positive; the other seven species (beaver, Chinese ferret-badger, Chinese hare, Chinese muntjac, domestic cat, hog-badger, and raccoon dog) sampled were negative.^12^

MERS-CoV was first found in 2012 in a lung sample from a 60-year-old patient who died of respiratory failure in Jeddah, Saudi Arabia. On 15 September 2012, a similar type of virus named human coronavirus was isolated from a patient with severe respiratory infection. Cases have also been reported in other countries.^18^ MERS has a 35% mortality rate, and since it emerged in the human population in June 2012, it has caused substantial morbidity and mortality.^19,20^ From 2012 until January 15, 2020, the total number of laboratory-confirmed MERS-CoV infection cases reported globally to the World Health Organization (WHO) was 2506, with 862 associated deaths, covering 27 countries (www.who.int/csr/don/31-january-2020-mers-united-arab-emirates/en). Cases of MERS from other countries were linked to travel to the Middle East.^20,21^ MERS-CoV was identified from the saliva of a patient with acute pneumonia and renal failure in Jeddah (KSA). MERS-CoV can be detected in respiratory tract secretions, as well as in feces, serum, and urine.^22–24^ In addition, it has been isolated from environmental objects such as bedsheets, bedrails, intravenous fluid hangers, and X-ray devices.^25^ In the case of known camel infection, MERS-CoV was transmitted from a camel to a human, which was confirmed by RNA sequencing.^26^

At the end of December 2019, the first clusters of patients with pneumonia caused by SARS-CoV-2 were reported in Wuhan, China.^27^ The basic reproduction number of SARS-CoV-2 is approximately 2.2, indicating that each patient would on average spread the infection to 2.2 people.^28^ Human-to-human transmission of SARS-CoV-2 occurred rapidly, and the atypical symptoms during the early stage may be a further disadvantage.^29^ Moreover, human infection might have begun months prior to the official announcement of the outbreak.^30^

To prevent further spread, stricter screening and surveillance are needed at travel hubs.^31^ Early detection, diagnosis, and treatment are also effective measures to contain the epidemic.^32^ Nonetheless, the fatality rate of SARS-CoV-2 is lower than that of SARS,^33^ with an estimated mortality risk of 2%.^34^ SARS-CoV-2 was first isolated from clinical specimens using human airway epithelial cells and the Vero E6 and Huh-7 cell lines.^27^ Approaches to assess virions include isolation from lower respiratory tract specimens^35^ and artificially infected specific pathogen-free human airway epithelial cells.^36^ Fluorescence quantitative PCR with primers designed according to specific sequences and serological testing aimed at IgG and IgM can be used to detect the virus.^9^

SARS-CoV, MERS-CoV, and SARS-CoV-2 pose major challenges to global health, causing infections in large parts of the world. SARS-CoV was found in 30 countries and MERS-CoV in 27 countries, while SARS-CoV-2 was found in 213 countries by 8th May, 2020. Details on the pandemics caused by SARS-CoV-2 are shown in Box 1, highlighting the status of the coronavirus-caused diseases worldwide.

Box 1 Epidemic information of COVID-19 As of May 8, 2020, there are 1,654,345 confirmed cases and 152,179 deaths in the European region; 1,586,129 confirmed cases and 87,930 deaths in the region of the Americas; 237,323 confirmed cases and 8608 deaths in the Eastern Mediterranean region; 157,447 confirmed cases and 6394 deaths in the Western Pacific region; 86,294 confirmed cases and 3075 deaths in the South-East Asia region; 33,973 confirmed cases and 1202 deaths in the Africa region.
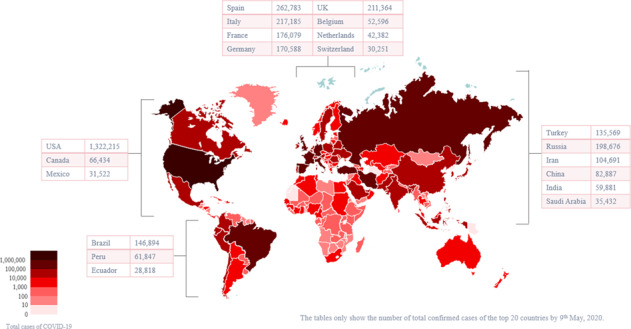

### Epidemiological analysis and symptoms of SARS, MERS, and coronavirus disease 2019 (COVID-19)

The clinical manifestations of SARS include hypoxia, cyanosis, high fever (above 38 °C), accelerated breathing or respiratory distress syndrome, and shortness of breath. X-rays show changes to the lungs to varying degrees. The WHO case definition (2003) includes the following: (1) fever higher than 38 °C or history of such in the past 2 days, (2) radiological evidence of new infiltrates consistent with pneumonia, (3) chills, cough, malaise, myalgia, or known history of exposure, and (4) positive test for SARS-CoV by one or more assays.^37^

Similar to SARS-CoV infection, MERS-CoV infection manifests as a severe lower respiratory tract infection with extrapulmonary involvement and high fatality rates.^19^ Symptomatic patients may present fever, chills, stiffness, myalgia, discomfort, cough, shortness of breath, and gastrointestinal symptoms of diarrhea, vomiting, and abdominal pain. Pneumonia is common, and severe infection with acute respiratory failure, renal failure, and shock is particularly frequent among older patients.^20^ Previous studies have estimated that approximately 12.5–25% of MERS-CoV infections may be asymptomatic.^20,38^ It must be noted that immunocompromised people are at high risk of MERS-CoV infection.^21^

For COVID-19, fever, cough, myalgia, and fatigue are most commonly observed at illness onset; less commonly observed are sputum production, hemoptysis, and headache, among others.^29,35^ Similar to SARS and MERS, some patients develop acute respiratory distress syndrome (ARDS).^39^ Around 81% of infected patients only develop mild symptoms, with mild pneumonia or no pneumonia. 14 and 5% are severe and critical status, respectively.^40^ Patients requiring ICU care tend to be much older and are more likely to have underlying comorbidities, such as hypertension and diabetes.^29^ Additionally, symptoms such as pharyngeal pain and anorexia are reported at higher rates among those admitted to the ICU than among non-ICU patients.^29^

Histopathologically, symptoms such as diffuse alveolar damage (DAD), loss of cilia, squamous metaplasia, denudation of bronchial epithelia, and giant-cell infiltrate often occur in the lungs of patients with SARS. The number of macrophages increases prominently in the alveoli and the interstitium.^41^ According to the morphological changes, observed, most authors have subclassified lung lesions in SARS into two or three consecutive phases: an acute exudative inflammatory phase, a fibrous proliferative phase, and a final fibrotic stage, although there is considerable overlap in histological findings among these phases.^42^ The main features of the first phase include extensive hyaline membrane formation, edema, alveolar hemorrhage, and fibrin exudation in alveolar spaces. The second phase includes widening of septae, pneumocyte hyperplasia, and organizing fibromyxoid and cellular exudates.^42,43^ The third phase includes septal and alveolar fibrosis.^42^ Several autopsies of SARS patients have demonstrated secondary bronchopneumonia, and the pathogens identified mainly consist of *Staphylococcus aureus, Mucor sp., Aspergillus sp., Pseudomonas aeruginosa*, and cytomegalovirus.^43^ SARS-CoV also greatly affects the immune system. For example, hemorrhagic necrosis is usually obvious in the lymph nodes and spleen.^44^ As described above, SARS-CoV attacks immune cells, especially T cells and macrophages, with approximately 50% of lymphocytes and 30% of monocytes in the circulation becoming infected.^42,45^ Under the influence of the virus, the patient’s germinal centers disappear, and both T and B lymphocytes are depleted. In the spleen, the white pulp shrinks, hemorrhage and necrosis occur in the red pulp, and the periarterial lymphatic sheaths decrease.^42,45,46^ With regard to the immune system, SARS-CoV can infect the lymphoid component of the intestine. For this reason, the patient’s submucosal lymphoid tissue atrophies.^47^ SARS-CoV can also infect epithelial cells in the mucosa of the small and large intestines, and the digestive tract may undergo mild diffuse inflammation and atrophy of the submucosal lymphoid tissues.^47^ According to previous reports, symptoms in the liver include extensive hepatocyte mitosis, balloon degeneration of hepatocytes, mild to moderate lymphocytic infiltrates, fatty degeneration, and central lobular necrosis.^42^ Additionally, apoptosis of liver cells has been confirmed in some cases.^48^ On autopsy, the kidneys of some SARS patients showed focal bleeding and acute tubular necrosis of different degrees. One of the major complications of ARDS is acute kidney injury.^42,49,50^ In many other cases, the features are nonspecific, including benign hypertensive nephrosclerosis and autolysis.^42^ Moreover, researchers have detected viral particles and genome sequences in the cytoplasm of neurons of the hypothalamus and cortex in the brain,^51^ which suggests that the virus can cross the blood–brain barrier and cause degeneration and necrosis of neurons, edema, extensive glial cell hyperplasia, and cellular infiltration.^42^

Pneumocytes and epithelial syncytial cells are important targets of MERS-CoV. The lung tissue presents DAD. Severe peripheral vascular diseases, patchy cardiac fibrosis, and hepatic steatosis have also been found in other organs.^52^ Another symptom is bronchial submucosal gland necrosis.^52^ Renal biopsy may show acute tubulointerstitial nephritis and acute tubulosclerosis with protein casts.^22^

In COVID-19 patients, a bilateral (sometimes unilateral) distribution of patchy shadows and ground glass opacity in the lungs is typical, based on CT scans.^29^ Plasma concentrations of interleukins (ILs) 2, 7, and 10, granulocyte-colony stimulating factor, interferon(IFN)-γ-inducible protein 10, monocyte chemoattractant protein 1, macrophage inflammatory protein 1α, and tumor necrosis factor α are increased in most dying patients.^39,53^ In addition, neutrophilia related to cytokine storms induced by virus invasion, coagulation activation related to a sustained inflammatory response, and acute kidney injury related to the effects of the virus, hypoxia, and shock, may be involved in mortality.^29^ Those who survive intensive care may experience long-term lung damage and fibrosis due to aberrant and excessive immune responses.^39^ Moreover, researchers found that as SARS-CoV-2 spread and changed across generations, clinical symptoms started to differentiate those infected previously from those infected more recently. The initial symptoms are becoming more insidious, which indicates that the virus may gradually evolve into an influenza-like virus and lie dormant for longer in asymptomatic patients.^54^

**Fig. 2:**
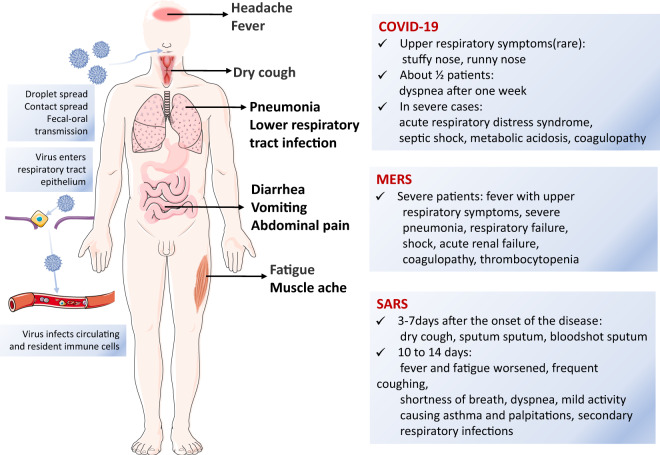
Common transmission methods and symptoms of coronavirus infection. The main modes of transmission include droplet transmission, contact transmission, and fecal–oral transmission. Fever, cough, fatigue, muscle soreness, and abdominal pain are common symptoms of the three coronavirus infections

In conclusion, in terms of outbreak times, SARS-CoV was the earliest, followed by MERS-CoV, then SARS-CoV-2. To date, MERS-CoV has the longest duration of infection, and SARS-CoV-2 appears to be the most infectious. SARS-CoV, MERS-CoV, and SARS-CoV-2 infections have similar symptoms, including fever, cough, myalgia, and shortness of breath, among others. The common transmission methods and symptoms of the three coronavirus infection are shown in Fig. 2.

### Structural studies of SARS-CoV, MERS-CoV, and SARS-CoV-2

**Fig. 3:**
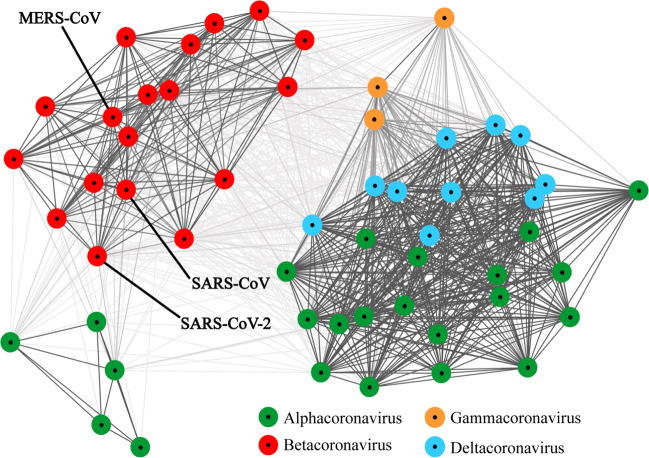
Clustering of the spike protein of each coronavirus. Fifty-four spike protein sequences filtered from each coronavirus coding sequences were clustered using the CLANS (CLuster ANalysis of Sequences) program on the website of MPI Bioinformatics Toolkit. Each colored dot represents the spike protein sequence of each coronavirus. Dots in the same color mean they are of the same genus, and each line shows the similarity of two sequences, with darker lines indicating higher similarity (lower *E* values). The Coronaviridae family includes the following genera: Alphacoronavirus (colored in green); Betacoronavirus (red); Gammacoronavirus (orange), and Deltacoronavirus (blue). The indicated SARS-CoV, MERS-CoV, and SARS-CoV-2 belong to the genus of Betacoronavirus

SARS-CoV is a coronavirus with a diameter of approximately 100 nm. Coronaviruses are the largest +ssRNA viruses and contain at least 14 ORFs,^16,55^ among which five main ORFs encode the replicase (Rep), and the S, E, M, and N proteins.^56^ Similar to other coronaviruses, there is a wide array of spike proteins on the envelope of SARS-CoV that resemble a corona. With its unique RNA-dependent RNA polymerase (RdRp), SARS-CoV often switches template strands during replication. Thus, if a cell is infected with several coronaviruses, RNA recombination may occur. The N protein combines with viral RNA to form a nucleocapsid, which is involved in the replication of SARS-CoV and is the most abundant protein in virus-infected cells.^56,57^ The M protein is a membrane glycoprotein that is involved in budding and envelope formation. The S protein of SARS-CoV is a type I transmembrane (TM) glycoprotein that is responsible for viral binding, fusion and entry into cells, as well as antibody induction.^16,58^ The S protein contains a signal peptide (SP: amino acids 1–12) and three domains called the extracellular domain (amino acids 13–1193), the TM domain (amino acids 1194–1215) and the intracellular region (amino acids 1216–1255).^16^ The extracellular domain consists of two subunits: S1 and S2. The fragment located in the middle region of the S1 subunit (amino acids 318–510) is the angiotensin-converting enzyme 2 (ACE2) receptor-binding region. The S2 subunit comprises a fusion peptide (FP) and two x 7-valent element repeats (HR1 and HR2) that are responsible for fusion between the virus and the target cell membrane.^58^ Thus, the S protein may be key to develop a vaccine to generate antibodies and block virus binding and fusion in the coronaviruses (Fig. 3).

The 3′ end of the SARS virus genome encodes 12 structural proteins and helper proteins, of which ORF3a, ORF6, ORF7a, and ORF7b have been proven to be viral structural proteins involved in the formation of viral particles. The 5′ end of the genome encodes 16 nonstructural proteins (NSPs), which are important for virus assembly, and may enable the design of small molecule drugs and/or vaccines.

MERS-CoV belongs to lineage C of the genus *Betacoronavirus* (*β*CoV) in the family *Coronaviridae*, order *Nidovirales*, and it is the first lineage C CoV and the sixth CoV known to cause human infection. MERS-CoV is an enveloped +ssRNA virus similar to other CoVs, but the amino acid sequence homology between MERS-CoV and other known CoVs is less than 90%.^19^ ORF1a and ORF1b located in the first two-thirds of the MERS-CoV genome are involved in virulence and encode NSPs (NSP1–16). The remaining third of the genome encodes four structural proteins, the S, E, M, and N proteins, as well as five accessory proteins (ORF3, ORF4a, ORF4b, ORF5, and ORF8b).^59–61^ The flanking regions of the genome contain the 5′ and 3′ untranslated regions (UTRs).^62^ Most MERS-CoV proteins are found in the cytoplasm.^63^ To date, the NS4b protein and the ORF3a-encoded protein are the only known coronavirus proteins to be detected in the nucleus.^64^ NSP1 suppresses host gene expression in infected cells, and its RNA cleavage-inducing function promotes virus assembly/budding.^65^ NSP16 is a viral 2′-*O*-methyltransferase (2′-*O*-MTase) that encodes critical functions in immune modulation and infection, suggesting that NSP16 is a conserved universal candidate for rapid design of a live attenuated vaccine.^66^ ORF4a’s protein can mask viral dsRNA to play a role in immune evasion, including type 1 IFN and protein kinase R-mediated antiviral stress responses.^67^ MERS-CoV NSP13 is a full-length coronavirus helicase that contains multiple domains, including an N-terminal Cys/His rich domain with three zinc atoms, a beta-barrel domain and a C-terminal superfamily (SF) 1 helicase (SF1) core with two RecA-like subdomains. This protein might interfere with the nonsense-mediated mRNA decay pathway to avoid degradation of viral RNA.^68^

The MERS-CoV S protein can recognize dipeptidyl peptidase 4 (DPP4) on the cell surface, promoting the fusion of the viral envelope and the cell membrane. The MERS-CoV S protein, a 1353-amino acid type I TM glycoprotein located on the viral envelope surface, is composed of S1 and S2 subunits.^69^ The structure of the S protein consists of four parts: an SP located at the N terminus (amino acids 1–12), an extracellular domain (amino acids 13–1195), a TM domain (amino acids 1196–1215), and an intracellular domain (amino acids 1216–1255).^16^ The S1 subunit contains two independent domains, an N-terminal domain (NTD, amino acids 18–353)^70^ and a C-terminal domain (amino acids 367–588), both of which may function as receptor-binding domains (RBDs);^71^ these domains serve as critical targets for the development of vaccines and therapeutics^72,73^ and facilitate the involvement of the S1 subunit in cell receptor (DPP4) binding. The core structure of the MERS-CoV S protein RBD is a five-stranded antiparallel sheet with several short helices, in which three disulfide bonds stabilize the core by connecting C383 to C407, C425 to C478, and C437 to C585.^16,73^ The accessory subdomain of the C-domain is a hypervariable region for receptor recognition and is called the receptor-binding motif (RBM). The accessory subdomains in SARS-CoV and MERS-CoV differ, resulting from divergent evolution and leading to the use of different receptors.^71^ DPP4 consists of an N-terminal hydrolase and a C-terminal β-propeller domain composed of eight lancets^74^ and the RBD. The S2 subunit of MERS-CoV contains an FP (residues 943–982), an HR1 domain (residues 984–1104), an HR2 domain (residues 1246–1295), a TM domain (residues 1296–1317), and an intracellular domain (residues 1318–1353).^69^ The S2 subunit is responsible for fusion between the virus and the target cell membrane. After the S1 subunit binds to DPP4, the S2 subunit inserts its FP into the target cell membrane to change its conformation. Its HR1 helix then forms a homotrimer with an exposed surface that has three highly conserved hydrophobic grooves, and binds with HR2 to form a six-helix bundle (6-HB) core structure, which facilitates fusion by bringing the viral and cell membranes into close proximity.^69^ The genetic material of MERS-CoV then enters the host cell via the fusion pore.^69^

SARS-CoV-2, which causes COVID-19, is a spherical betacoronavirus with a diameter of 60–140 nm and unique spikes ranging from 9 to 12 nm.^27^ Its RNA has the typical betacoronavirus organization, containing a 5′ UTR, a gene encoding the replicase complex (ORF1a/b), the S gene, E gene, M gene, and N gene, a 3′ UTR, and several unidentified nonstructural ORFs.^27,75^ The length of the sequences is 265 nt for the 5′ terminal region, 229 nt for the 3′ terminal region, 3822 nt for the S gene, 228 nt for the E gene, 669 nt for the M gene, and 1260 nt for the N gene.^75^ The 21,291-nt-long ORF1a/b gene contains 16 predicted NSPs.^75^ Similar to SARS-CoV, there is a predicted ORF8 gene that is 366 nt in length and located between the M and N ORF genes.^75^ Nucleotides 1, 1029, and 1652 may be the recombination breakpoints because based on the fragments from nt 1 to nt 1029 and nt 1652 to the end, SARS-CoV-2 seems to be related to bat-SL-CoVZC45 and bat-SL-CoVZXC21; however, when considering the region from nt 1030 to nt 1651, SARS-CoV-2 is mostly likely to be grouped with SARS-CoV and bat SARS-like CoVs. More research is required to analyze the recombination of the genome as a whole.^75^ With over 10% of its conserved replicase complex being different from those of other betacoronaviruses, SARS-CoV-2 solely occupies a newly created subclade within the *Sarbecovirus* subgenus.^27,75^ Bats may be its natural host, but owing to several arguments including the complicated environment in the wet markets in Wuhan, this remains unclear, and further research is needed.^36,75–77^ The free energy of the spike protein of SARS-CoV-2 is much lower than that of SARS-CoV, indicating that SARS-CoV-2 is more stable than SARS-CoV.^78^ The high similarity of the RBD sequences and of the spike protein structures between SARS-CoV-2 and SARS-CoV,^36,75,79^ also the simulation of the spike protein of SARS-CoV-2 binding to ACE2,^80^ the receptor of SARS-CoV, and results shown that ACE2 plays a vital role in SARS-CoV-2 entry into HeLa cells,^76^ indicating that SARS-CoV-2 also uses ACE2 for cell entry. Structural modeling of the ACE2-B0AT1 complex (B0AT1 is used to obtain stable ACE2) suggests that the complex can bind two S proteins simultaneously, providing important clues to the molecular basis of coronavirus recognition and infection.^81^ The RBD-ACE2 binding free energy for SARS-CoV-2 is significantly lower than that for SARS-CoV, in accordance with the fact that SARS-CoV-2 is more infectious than SARS-CoV.^78^ A recent study found that CD147, a kind of TM glycoprotein, facilitates cell entry of SARS-CoV-2 functionally, and its affinity constant with the S protein is 1.85 × 10^−7^ M.^82^ Table 1 shows a comparison of the structures of SARS-CoV, MERS-CoV, and SARS-CoV-2. The specific function of the SARS-CoV-2 proteins, including S, E, M, and N proteins, need further study.

**Table 1:** The comparison of the structural proteins of SARS-CoV, MERS-CoV, and SARS-CoV-2

Virus	Structural proteins								References
SARS-CoV	S protein	S1 subunit	The RBD (residues 318–510)	Cell receptor (ACE2) binding	ACE2	Comprises 805 amino acids and contains a single catalytic domain			^58^
			The RBM (residues 424–494)						
		S2 subunit	FP	The fusion of virus and target cell membrane					
			HR1 (residues 892–1013)						
			HR2 (residues 1145–1195)						
		N protein		Combines with viral RNA to form a nucleocapsid					^56^
		M protein		Play important roles in viral assembly					
		E protein							
MERS-CoV	S protein	S1 subunit	The RBD (residues 367–588)	Cell receptor (DPP4) binding	DPP4	N-terminal hydrolase			^68,69,70,71^
			The RBM (residues 484–567)						
		S2 subunit	FP (residues 943–982)	The fusion of virus and target cell membrane					
			HR1 (residues 984–1104)						
			HR2 (residues 1246–1295)			C-terminal β-propeller domain			
			Transmembrane domain (residues 1296–1317)						
			Intracellular domain (residues 1318–1353)						
		N protein		Binds to the RNA genome to form a nucleocapsid					
		M protein		Is important in viral assembly, the formation of the viral envelope, and the formation of the viral core					
		E protein		Plays an important role in intracellular trafficking, host recognition, viral assembly, and virus budding					
SARS-CoV-2	S protein	S1 subunit	The RBD	Cell receptor (ACE2) binding	ACE2	comprises 805 amino acids and contains a single catalytic domain			^78–80^
			The RBM						
		S2 subunit	FP	The fusion of virus and target cell membrane					
			HR1						
			HR2						
			Transmembrane domain						
			Connector domain						
		N protein		Combines with viral RNA to form a nucleocapsid					^75^
		M protein		Play important roles in viral assembly					
		E protein							

In conclusion, SARS-CoV, MERS-CoV, and SARS-CoV-2 are similar in structure. Their major proteins are structural proteins, including the S, E, M, and N proteins, accessory protein, and NSPs (NSP1–16). All these viruses rely on the S protein to identify cell targets and fuse with membranes. Their S proteins are composed of S1 and S2 subunits. The S1 subunit contains an NTD and a C-domain, in which there is an RBD. The accessory subdomain of the C-domain is a hypervariable region for receptor recognition and is called the RBM. The difference is that SARS-CoV recognizes ACE2, SARS-CoV-2 recognizes ACE2 and CD147, but MERS recognizes DPP4, which results from the difference between the accessory subdomains of SARS-CoV, SARS-CoV-2, and MERS-CoV. The function of the accessory and nonstructural proteins of these three viruses is related to viral replication and assembly, hence linked to virulence.

### Pathogenesis of SARS-CoV, MERS-CoV, and SARS-CoV-2

SARS-CoV and SARS-CoV-2 are nearly identical in terms of invasion and self-replication, whereas MERS-CoV has different targets. The cellular receptor of SARS-CoV and SARS-CoV-2 is ACE2, plus CD147 for SARS-CoV-2, while that of MERS-CoV is DPP4. The human ACE2 protein is a typical zinc metallopeptidase that comprises 805 amino acids and contains a single catalytic domain. It is a type I integral membrane glycoprotein that is oriented with an extracellular N terminus and a catalytic site that functions in metabolizing circulating peptides.^83^ It is believed that there is a virus-binding hot spot on ACE2 that is not pre-existent or preorganized but is induced to form upon virus binding.^84^ The hot spot consists of a salt bridge surrounded by hydrophobic tunnel walls. The general structural features of the hot spot favor virus binding: it is located in a region on ACE2 that is furthest from the membrane, relatively flat, and free of glycosylation and is thereby easily accessible to viruses. The hot spot is mainly an intrinsic property of ACE2, but it is also a dynamic structure and receives structural contributions from both ACE2 and the viruses, with the contributions of ACE2 being more pronounced. DPP4 is a type II transmembrane glycoprotein that consists of approximately 766 amino acids. DPP4 contains an N-terminal hydrolase and a C-terminal β-propeller domain composed of eight lancets.^74^ The S protein RBD binds to the side surface of the DPP4 propeller and the amino acid residues in lancets 4 and 5, including K267, Q286, T288, R317, R336, Q344, A291, L294, and I295.^74^

The pathogenic mechanism of SARS-CoV is believed to include two parts: the virus injures target cells directly, and subsequent immune system dysfunction leads to indirect injury.^42^ SARS-CoV spreads via droplet and contact transmission and via the fecal–oral route. Through droplet inhalation, the virus enters the respiratory tract and invades epithelial cells. Infection and replication of the virus and local inflammatory changes contribute to lung tissue damage. Subsequently, SARS-CoV infects circulating and resident immune cells. The key cells in this step consist mainly of T cells and macrophages. The viruses are then carried to other organs, including secondary lymphoid organs, by these circulating immune cells.^42^ SARS-CoV resembles HIV because both viruses attack immune cells and cause immunodeficiency.^42^

SARS-CoV causes lung injury mainly by inhibiting the function of ACE2. The virus binds to ACE2 via the spike protein, downregulating its function and contributing to lung injury. ACE2 is an important component of the renin–angiotensin system (RAS). In this system, angiotensinogen is first converted to angiotensinI (AngI) by renin, and then AngIis converted to Ang II under the influence of ACE2. ACE2 downregulates the levels of AngI and Ang II, which can bind to the Ang II type I (AT1) receptor and cause certain types of lung injury, mainly pulmonary hypertension, pulmonary fibrosis, and acute lung injury.^85^ Ang II can directly induce pulmonary artery smooth muscle cells to grow or proliferate rapidly through AT1, thus causing pulmonary hypertension.^86^ Ang II contributes to pulmonary fibrosis by promoting the expression of the profibrotic cytokine transforming growth factor-b1, causing fibroblasts to convert to myofibroblasts and accumulate collagen.^70^ Several strategies can be used by the virus to escape the innate immune response. Normally, viral pathogen-associated molecular patterns (PAMPs) are detected by host pattern recognition receptors (PRRs). PAMPs include viral dsRNA and mRNA, and PRRs include retinoic acid-inducible gene I protein (RIG-I) and melanoma differentiation-associated protein 5 (MDA5). Production of type I IFN is induced via the nuclear factor-κB (NF-κB) pathway. When IFN binds to the IFNα/β receptor, signal transducer and activator of transcription (STAT) proteins are activated, which increases the production of other antiviral proteins and thus blocks SARS-CoV replication for further infection.^11^ Additionally, SARS-CoV could encode several proteins that hinder the signaling cascades downstream of PRRs.

The cell receptor of MERS-CoV is DPP4, which is widely expressed on epithelial cells in the prostate, alveoli, kidneys, liver, and small intestine and on activated leukocytes; thus, the range of MERS-CoV tissue tropism is broader than that of any other similar coronavirus. MERS-CoV infection of dendritic cells and macrophages can lead to the continuous production of proinflammatory cytokines and chemokines (such as TNF-α, IL-6, CXCL-10, CCL-2, CCL-3, CCL-5, and IL-8).^87^ Compared to SARS-CoV infection, MERS-CoV infection can cause higher expression levels of IL-12, IFN-γ, IP-10/CXCL-10, MCP-1/CCL-2, MIP-1α/CCL-3, RANTES/CCL-5, and IL-8.^87^ Induction of immune cell-recruiting cytokines/chemokines might lead to the infiltration of a large number of immune cells into the lower respiratory tract, causing severe inflammation and tissue damage.^87^ MERS-CoV can also infect T cells and lead to apoptosis, avoiding the host immune response and facilitating faster spread.^88^

MERS-CoV has also evolved a mechanism to escape the host cell immune system. When host sensors initiate signaling pathways, transcription of type I IFN genes begins, and the type I IFN response, which is an essential component of antiviral innate immunity, is initiated.^89–91^ IFN activates the transcription of numerous IFN-stimulated genes (ISGs) and synthesizes 2′,5′-oligoadenylate (2-5A), which causes RNase L activation.^91–93^ Activated RNase L can cleave both viral and host ssRNA, leading to translation stagnation and apoptosis and limiting virus replication and transmission in vitro and in vivo.^92,94^ However, the MERS-CoV protein NS4b has phosphodiesterase activity and antagonizes RNase L via enzymatic degradation, leading to inference with IFN signaling. Although NS4b is mainly expressed in the nucleus, the expression level of the NS4b protein in the cytoplasm is sufficient to prevent activation of RNase L.^88^ In addition, the MERS-CoV M, ORF4a, ORF4b, and ORF5 proteins are reported to be strong IFN antagonists, among which the ORF4a, ORF4b, and ORF5 proteins inhibit both type I IFN induction^61,95,96^ and NF-κB signaling pathways, which are similar to the type I IFN signaling pathway,^96^ providing a possible mechanism for MERS-CoV evasion of innate immunity.^88,96^

For SARS-CoV-2, the first cluster of patients was believed to be associated with a seafood market.^27^ However, due to the identification of patients without direct exposure to the market, transmission between humans was proven.^97^ Hospital-related transmission has also been detected via infected medical workers and hospitalized patients. Main routes of transmission include respiratory droplets, close contact, and extended exposure to aerosol environments loaded with high concentrations of virions. In addition to transmission through the respiratory tract, exposure of unprotected eyes to SARS-CoV-2 might cause acute respiratory infection.^98^ Notably, the virus can be transmitted during the incubation period.^99^

The TM protease serine type 2 (TMPRSS2) primes the S protein of SARS-CoV-2 for cell entry.^100^ Based on analysis of the ACE2 RNA expression profile in normal human lungs, SARS-CoV-2 mainly infects type II alveolar (AT2) cells (only 0.64% of human lung cells can express ACE2, 83% of which are AT2 cells), and they express many other genes that facilitate SARS-CoV-2 infection;^101^ moreover, CD147 was also reported that it probably functionally facilitates cell entry of SARS-CoV-2.^82^

High expression of ACE2 has also been detected in cells of the digestive system, such as upper esophageal cells; accordingly, this system is a potential route of infection.^102^ In addition to the lungs and intestines, other organs, such as the heart, esophagus, kidney, bladder, testis, ileum, and adipose tissue, also express ACE2, and the expression level is higher than that in the lungs.^103,104^ Certain tumor tissues have higher expression of ACE2, making cancer patients more vulnerable than other people.^104^

**Fig. 4:**
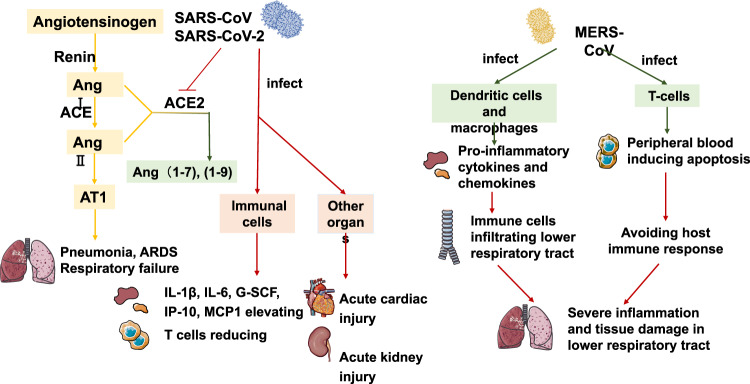
Pathogenesis of SARS-CoV, MERS-CoV, and SARS-CoV-2. SARS-CoV and SARS-CoV-2 play a pathogenic role by inhibiting ACE2. Under the influence of renin and ACE, angiotensinogen is converted into Ang II. Through the AT1 receptor, Ang II acts as a lung injury-promoting factor, and in some cases, may cause vascular constriction, an inflammatory response, cell proliferation, fibrosis, and apoptosis of alveolar epithelial cells, resulting in diseases such as pulmonary hypertension, pulmonary fibrosis, and acute lung injury. ACE2 converts Ang II into Ang (1–7), and through the MAS receptor, Ang (1–7) play roles in vasodilation, antiproliferative activity, and antioxidant activity. SARS-CoV downregulates the activity of ACE2 and causes an increase in the amount of Ang II and lung injury. MERS-CoV infection of dendritic cells and macrophages can lead to the continuous production of pro-inflammatory cytokines and chemokines, leading to a large number of immune cells infiltrating a patient’s lower respiratory tract, causing severe inflammation and tissue damage. MERS-CoV can infect T-cells from human lymphoid organs and causes the peripheral blood inducing apoptosis by intrinsic and extrinsic pathways, thus avoiding host immune response

In conclusion, SARS-CoV and SARS-CoV-2 bind cells expressing ACE2. They affect the RAS system due to the affinity for ACE2 (much stronger with SARS-CoV-2 than with SARS-CoV), leading to the onset of symptoms. MERS-CoV causes symptoms by producing inflammatory cytokines and invading T cells. The specific pathogenic mechanisms of these three viruses are illustrated in Fig. 4.

## Diagnosis and therapy

### Diagnostic methods

In addition to clinical manifestations, definitive diagnosis and confirmation of a coronavirus infection requires specific testing. The criteria for the diagnosis of SARS from the WHO include (1) fever higher than 38 °C or history of such in the past 2 days, (2) radiological evidence of new infiltrates consistent with pneumonia, (3) chills, cough, malaise, myalgia, or known history of exposure, and (4) positive tests for by at least one assay. As for MERS, the criteria for the diagnosis according to the WHO include (1) fever, cough, and hospitalization with suspicion of lower respiratory tract lesion, (2) history of contact with probable or confirmed cases, (3) history of travel or residence within the Arabian Peninsula, and (4) positive tests by a PCR-based detection method using respiratory samples, such as nasopharyngeal swabs, nasopharyngeal or tracheal aspirates, bronchoalveolar lavage (BAL) and induced sputum, or by serological tests such as the rapid immunochromatographic assay using highly selective monoclonal antibodies at room temperature. The initial criteria for the diagnosis of SARS included (1) a history of travel or residence in Wuhan within 2 weeks before the onset of illness, (2) exposure to patients from Wuhan with fever and respiratory symptoms within 2 weeks before the onset of illness or the existence of clusters of diseases, (3) fever accompanied by radiographic features of pneumonia, a normal or decreased total number of white blood cells, or a decreased lymphocyte count, and (4) fluorescence PCR testing positive for nucleic acids of SARS-CoV-2.^37,59,105–107^

Early diagnosis of MERS is difficult, but several highly specific and sensitive molecular and serologic assays exist for diagnosis in both animals and humans.^108^ Among them, a rapid immunochromatographic assay can detect MERS-CoV antigens based on the detection of the MERS-CoV nucleocapsid protein in a short time frame; the relative sensitivity and specificity of this test are 93.9 and 100%, respectively.^105^

Nucleic acid tests, such as real-time PCR tests, were mostly used to detect virus in the early stage of the outbreak of SARS-CoV-2, although this approach requires a relatively long time and is not conducive to accelerating diagnosis or large-scale application. Therefore, alternative, rapid diagnostic kits for SARS-CoV-2 may be used. For example, the Colloidal Gold Detection Kit uses serum, plasma, or whole blood samples to detect IgM/IgG antibodies for diagnosis on the 7th day of infection or the 3rd day after onset and requires only 15 min. The nucleic acid detection kit for six respiratory viruses, including SARS-CoV-2 and influenza A virus, uses thermostatic amplifier chips for diagnosis. By detecting different infections, it may help distinguish people with influenza from those with other viral infections. A newly developed detection method, Nanopore Targeted Sequencing, also has the potential for efficiently detecting viruses in a reasonable time. Moreover, it could identify 40 other respiratory viruses and monitor changes in virulence and transmissibility caused by viral mutations.^109^

### Therapeutic agents

All patients should be treated in isolation.^53^ Based on previous studies, drugs may be developed to inhibit cell entry. Several strategies have been investigated to identify specific antivirals targeting the S protein, including MERS-RBD-targeted nAbs that block viral attachment, peptide inhibitors targeting S2 to prevent the formation of the fusion core, and small molecules without defined mechanisms.^110^ sDPP4 has been found to bind MERS-CoV RBD with high affinity, suggesting that sDPP4 could serve as a blocker for MERS-CoV attachment to and entry into cells.^111,112^ One study reported a monoclonal antibody, 7D10, that binds to the NTD to inhibit cellular entry of MERS-CoV.^113^ Experiments have shown that 7D10 not only inhibits the binding of MERS-CoV to cells but also has effects after viral-cell attachment. 7D10 inhibits virus invasion by blocking the binding of the S1 subunit to the receptor and interfering with the conformational transformation of the S2 subunit when the virus fuses with the cell membrane.^113^ A peptide fragment spanning residues 736–761 of the S protein exerts neutralization activity by inhibiting membrane fusion and the entry of MERS-CoV, suggesting that it is possible to develop vaccines targeting this neutralizing epitope.^114^ Griffithsin, a lectin derived from red algae, binds to oligosaccharides on the surface of SARS-CoV S protein and may also prove effective against SARS-CoV-2.^34,115^ Researchers found that antibodies raised against SARS-CoV, such as CSS-2, -3, -4, and -5, could cross-neutralize SARS-CoV-2 by reducing S-driven cell entry, although the efficiency was lower than that observed for SARS-CoV.^100^ Meplazumab, an anti-CD147 humanized antibody, binds with the CD147 in competition with the S protein,^116^ thus significantly inhibiting virus from invading cells and reducing the time of negative conversion.^82,106^ Chloroquine could effectively inhibit SARS-CoV-2 in vitro, and hydroxychloroquine was even more potent than chloroquine.^117,118^ Hydroxychloroquine could significantly help reduce the virus load, and azithromycin could reinforce its effect.^119^ Arbidol could also contribute to condition improvement.^120^ Note that these studies were generally limited and further trials and development are necessary.

Protease inhibitors also have the potential for coronavirus therapeutics. Protease inhibitors, including disulfiram, lopinavir, and ritonavir, have been reported to be effective against SARS and MERS, and disulfiram, a drug for alcohol dependence, was reported to inhibit the papain-like protease (PLpro) of MERS-CoV and SARS-CoV in cell cultures.^34,108^ In vitro, SARS-CoV can be inhibited by both 6-mercaptopurine and 6-thioguanine targeting PLpro.^121^ When used together with ribavirin, the protease inhibitors lopinavir and ritonavir have improved the outcomes of patients with SARS compared with those achieved with the use of ribavirin alone.^122^ Such inhibitors are also being tested in patients infected with SARS-CoV-2, but whether these inhibitors effectively suppress 3CLpro and PLpro of SARS-CoV-2 remains controversial.^34^ Drugs such as colistin, valrubicin, icatibant, bepotastine, epirubicin, epoprostenol, vapreotide, aprepitant, caspofungin, perphenazine, prulifloxacin, bictegravir, nelfinavir, and tegobuvir bind to the main protease of SARS-CoV-2, and thus, are potential candidates.^123^ Lianhua-Qingwen formula, a kind of traditional Chinese medicine, has been also recently described theoretically as a potential treatment candidate against the main virus protease.^124^ The serine protease inhibitor camostat mesylate could partly block SARS-CoV-2 infection of lung cells by inhibiting TMPRSS2.^100^

Viral RNA synthesis blockers also have potential. Remdesivir has broad-spectrum activity against MERS-CoV and SARS-CoV in cell cultures and animal models and is currently in clinical development for the treatment of Ebola virus disease.^34,125^ It was also reported positively based on a patient with SARS-CoV-2 infection in the US,^126^ and another study showed that it was able to inhibit the virus.^117^ Ribavirin is often used to treat SARS and MERS, but it is uncertain whether it is potent enough against COVID-19.^34,108^ Regardless, when used alone, ribavirin has minimal activity against SARS-CoV and may cause strong side effects. It is often used together with corticosteroids.^14,127^ A preclinical study of galidesivir (BCX4430), an adenosine analog, revealed antiviral activity against SARS-CoV and MERS-CoVCoV.^115^ A study of favipiravir (T-705), a guanine analog, proved its activity against SARS-CoV-2, and randomized trials of favipiravir plus interferon-α (ChiCTR2000029600), and favipiravir plus baloxavir marboxil (ChiCTR2000029544) are in progress.^34^

Corticosteroids may be used to treat SARS patients to suppress lung inflammation; although this therapy is not associated with mortality in patients with MERS, it is associated with delayed clearance of MERS-CoV RNA.^14,128^ Moreover, clinical evidence does not support the use of corticosteroids for COVID-19 treatment.^129^

Regarding antibodies, plasma therapy using convalescent plasma from fully recovered patients is effective against these coronaviruses, including SARS-CoV-2.^108,130^ Tocilizumab (anti-human IL-6 receptor) may curb immunopathology and trials are ongoing now.^131^ A study of novel monoclonal antibodies against the MERS-CoV spike protein suggests that mAbs can be utilized for the identification of specific mutations of MERS-CoV.^132^ Wang et al. provided an all-hydrocarbon-stapled peptide that likely mimics the native conformation of the C-terminal short α-helical region of the MERS-CoV S protein, which can block the formation of the hexameric coiled-coil fusion complex to inhibit viral-cell membrane fusion.^133,134^

Current treatments for SARS mainly include ribavirin, IFN α, plasma therapy, host-directed therapies, and systemic corticosteroids.^14,41^ Due to the lack of clinical trial data, adequate supportive care supplemented with different combinations of drugs remains the main treatment for patients.^14,41^

For MERS, despite many studies in humans, there is no consensus on the best treatment; thus, randomized clinical trials are needed to assess potential treatment options.^134^ Several therapeutics are in development, including convalescent plasma, lopinavir/ritonavir, ribavirin, IFN and novel therapies, including polyclonal antibodies and broad-spectrum antivirals.^108^ Antimicrobial peptides (AMPs) may be used as alternative therapeutic agents,^135^ and many have been effective even against bacterial proteases agents.^136^ Antiviral drugs with effective activity in vitro include neutralizing monoclonal antibodies, antiviral peptides, mycophenolic acid, lopinavir, IFN, ribavirin, nitazoxamide, mycophenolate mofetil (MMF), alisporivir, silvestro, and corticosteroids.^19,21,59^

Although there is no agreement on the most ideal treatment option to cure COVID-19, much research activities and pursued routes are underway.

### Potential host-targeted agents

The host-directed strategy is another approach for limiting viral replication. Host proteases such as cathepsin B and cathepsin L can cleave the spike protein of SARS-CoV. Drugs such as camostat inhibit host serine proteases and then interfere with the entry of SARS-CoV, but they may cause many significant side effects.^14,135^ Pegylated IFN α-2a and -2b may be used to stimulate innate antiviral responses in patients, and chloroquine, an approved immune modulator, was reported to have inhibitory effects against SARS-CoV-2 under certain conditions.^34^ Researchers have also proposed that some commercially available drugs with suitable safety profiles, such as metformin, glitazones, fibrates, sartans, and atorvastin, as well as nutrient supplements and biologics, might reduce immunopathology, boost immune responses, and prevent or curb ARDS. In addition, ongoing cellular therapies using mesenchymal stromal cells from allogeneic donors have been shown to reduce nonproductive inflammation and affect tissue regeneration and are being evaluated in phase 1/2 trials in patients with ARDS; these therapies may be assayed in SARS-CoV-2-infected patients.^40,137,138^ Expansion of antiviral T cells as cellular drugs could aid in preparing T cell products for adjunct treatment of patients with severe infection.^40^

Overall, there are similarities and differences among the treatments for these three coronaviruses. Notably, we summarize the potential drugs and vaccines in Table 2.

**Table 2:** Potential drugs and vaccines against three coronaviruses

		SARS-CoV	MERS-CoV	SARS-CoV-2	References
Potential virally targeted agents	Potential drugs inhibiting cell entry	CP-1 Griffithsin CSS-2,3,4,5	HR2P sDPP4 7D10	EK1 EK1C4 Griffithsin CSS-2,3,4,5	^34,100,111,113,115,164,165^
	nAbs	CR3022 m396 80R 33G4	m336 MERS-4 MERS-27	CR3022	^160–163^
	Protease inhibitors	Remdesivir Ribavirin Galidesivir Lopinavir Bananins 5-Hydroxychromone derivatives Disulfiram	Favipiravir Ribavirin Penciclovir Remdesivir Lopinavir Ritonavir Darunavir	Remdesivir Ribavirin Galidesivir Lopinavir Ritonavir Camostat Mesylate	^34,111,121,122^
	Viral RNA synthesis blockers		Remdesivir		^117^
		Ribavirin Galidesivir (BCX4430)		Favipiravir (T-705)	^14,34,41,108,115,122,126^
	Other agents		Convalescent plasma		^108^
		Corticosteroids 6-mercaptopurine-6-thioguaninet IFNα Antiviral treatments	IFNα Antimicrobial peptides (AMPs) Mycophenolic acid Nitazoxamide Mycophenolate mofetil (MMF)		^14,19,38,41,59,122,133,134^
Potential host-targeted agents		Pegylated interferon alfa-2a; Pegylated interferon alfa-2b; Metformin; Glitazones; Fibrates; Sartans; Atorvastin; nutrient supplements; biologics; cellular therapies; IFNα together with immunoglobulins or thymosins;			^34,39,136^
		Cathepsin B cathepsinL Camostat		Chloroquine	^14,34,100,134^
Spike-based vaccines		A recombinant fusion protein (designated RBD-Fc) containing 193-amino acid RBD and a human IgG1 Fc fragment	Simian adenovirus vector vaccine (ChAdOx1);an Ad vector encoding MERS-CoV S1 extracellular domain (Ad5.MERS-S1);an RABV vector encoding S1 elicit antibody;RBD-based vaccines		^61,138,139,140^

### Vaccines

Vaccination could be used to prevent infection or to reduce disease severity. Different kinds of vaccines, such as DNA vaccines, recombinant proteins, subunit vaccines, and inactivated viruses, were described.^14^ However, the highly sophisticated immune evasion mechanisms of viral pathogens and their high mutation rates make human vaccine development a major challenge.^136^ The four NSPs (3CLpro, PLpro, helicase, and RdRp), which are key enzymes in the viral life cycle, and the S protein, which is responsible for receptor binding during cell entry, are attractive targets for developing vaccines against coronaviruses.^115^ Among them, the S protein is most commonly targeted.^61^

Based on previous studies, it is believed that the S protein receptor-binding domain (S-RBD) located in the S1 subunit is an important target for the development of a SARS vaccine, especially the key neutralizing region, CND. Indeed, CND can induce a strong neutralizing antibody response and cross-protection against SARS-CoV mutants. One study showed that a recombinant fusion protein (designated RBD-Fc) containing the 193-amino acid RBD and a human IgG1 Fc fragment can induce highly potent antibody responses in immunized rabbits. The antibodies inhibited SARS-CoV infection (serum dilution of 1:10,240), and they are believed to be safer than other types of vaccines.^139^ Additionally, with chloroplast transgenic technology, it is possible to combine the fusion gene S-RBD and the carrier molecule cholera toxin B subunit into the tobacco chloroplast genome to obtain a chloroplast transgenic tobacco plant that stably expresses the oral SARS-CoV subunit vaccine.^140^ For MERS, vaccines based on the viral S protein include full-length S or S-trimer protein-based vaccines such as a full-length S-based simian adenovirus vector vaccine (ChAdOx1) and DNA vaccine (GLS-5300),^61^ S1-based vaccines such as an Ad vector encoding MERS-CoV S1 extracellular domain (Ad5.MERS-S1) and an RABV vector encoding an S1-elicited antibody,^141,142^ RBD-based vaccines, and vaccines based on other regions. It has been confirmed that the S proteins of SARS-CoV and SARS-CoV-2 are quite similar, but researchers recently found that the three monoclonal antibodies developed to bind to the S protein of SARS-CoV, S230, m396, and 80R do not cross-react with the S protein RBD of SARS-CoV-2, suggesting that tailored vaccines and antibodies against SARS-CoV-2 must be designed.^143^

DNA vaccines are also quite promising. Specific IgG antibodies against SARS-CoV can be promoted by the S gene DNA vaccine. As mentioned above, the S protein of SARS-CoV plays an important role during the pathogenic process, and synthetic peptides induced by DNA vaccine in *Escherichia coli* elicit specific antibodies against the SARS-CoV S protein which might provide another approach for further developing SARS-CoV vaccines.^42,144^ To evaluate the safety and immunogenicity of a plasmid DNA vaccine (GLS-5300) that expresses the S protein of MERS-CoV, a phase I clinical trial on healthy volunteers was conducted in 2016, but the results were not reported. Another phase I trial utilizing the viral vector, Chimpanzee Adenovirus, Oxford University #1 (ChAdOx1), containing the MERS-CoV S protein expression gene was started by Oxford University in January 2018.^145^

**Fig. 5:**
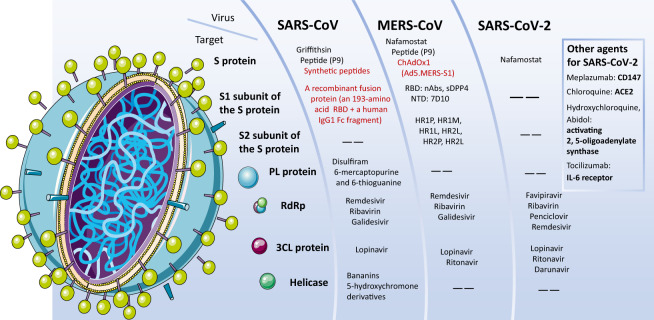
The targets of the different drug candidates against the three coronaviruses. Common targets against the three coronaviruses are mainly the S protein and the S1/S2 subunits, PL protein, RdRp, 3CL protein, and Helicase. The figure shows drug candidates (in black) and vaccines (in red). Among them, Remdesivir has been trending in the news recently. It inhibits the RdRp, is in phase III for SARS-CoV-2, and may have an effect on the three viruses. Ribavirin in combination with a pegylated interferon may also have an effect against the three viruses. Ritonavir and Lopinavir, which inhibit the 3CLpro and are in phase III for SARS-CoV-2, have an effect on both SARS-CoV-2 and MERS-CoV. DNA vaccines and vaccines based on the S protein or subunits of the S protein are in development

In addition, camel vaccines against MERS-CoV are a consideration. At present, at least two promising candidate camel vaccines are undergoing development, and field trial evaluation is in progress.^108,146^ One study found that the RBD fragment covering spike residues 377–588 is a key neutralizing receptor-binding fragment and an ideal candidate for MERS vaccines.^147^ Another potential neutralizing epitope is a peptide fragment covering 736–761 residues of the S protein which blocks the membrane fusion and cellular entry of MERS-COV^114^ (Fig. 5).

### Other approaches

Recombinant viruses may be employed to generate an immune response against the viruses. There are two kinds of recombinant viruses which are promising for designing a protective vaccine. The first is a defective or nonpathogenic vector capable of expressing viral proteins, and the second is a vector that can be assembled in a test tube to stimulate the assembly of virus-like particles.

Adenosine deaminase (ADA), a kind of human enzyme, could interact with DPP4, therefore, ADA could compete with MERS-CoV as a natural antagonist when the virus attempts to bind to cells.^148^ In addition, anti-DPP4 can play a similar role,^149^ however, it is not practical to use this method in vivo because DPP4 has important functions in the regulation of several different signaling pathways.^150^

## Perspectives and further directions

### Questions concerning the coronavirus–host interactions

This topic has intrigued the community. Understanding certain mechanisms about virus interactions will support drug research activities. For instance, it was found that SARS-CoV-2 has a stronger, more rapid spreading ability than many known viruses. Is it possible that it invades host cells via additional novel routes, not limited to ACE2? For example, CD147 is probably involved in virus invasion.^82^ Is there an S-like protein involved in invasion? The affinity of the human ACE2 protein for the RBD of the new coronavirus is 10–20 times higher than it is for that of the SARS virus, which likely explains why SARS-CoV-2 spreads more swiftly. There is a distinct insert present in the peptide fragment spanning of S1/S2 loop of the SARS-CoV-2 S protein, which is not shared the similarity with SARS-CoV or any SARS-related viruses. Does this peptide loop impact on the entry pathway type of SARS-CoV-2, compared to other known betacoronavirus lineage B? Furthermore, the S protein is likely cleaved during virus assembly and delivery to the cell surface by Golgi-resident proprotein convertases such as furin,^151^ which is different from the behavior of its close relatives. Furin is found in a variety of human tissues, including the lungs, liver, and small intestine. Therefore, which are exactly the cell types that SARS-CoV-2 may attack? Yet, in relation to the last two questions, it should be noted that studies are also necessary to investigate any possibility of receptor-independent virus entry.

### Potential targets and drugs against SARS-CoV-2

The SARS-CoV-2 genome encodes nonstructural proteins, structural proteins, and accessory proteins. 3CLpro, PLpro, helicase, and RdRp, which belong to the first type, and the S protein, which belongs to the second, have been recognized as promising targets for developing antiviral agents against SARS-CoV and MERS-CoV, which therefore may be applied to SARS-CoV-2. Nonetheless, the rapid identification of effective interventions against SARS-CoV-2 is a major challenge. However, the subject of using currently known antiviral drugs has been frequently brought up by the community as a potential short-term strategy to combat SARS-CoV-2.

Drugs affecting such targets and their status for SARS-CoV-2 are listed below. The main candidates as inhibitors of 3CLpro include lopinavir, ritonavir, darunavir, cobicistat, and ASC09F (phase III, in combination with oseltamivir, for SARS-CoV-2).^152^ Candidates as inhibitors of PLpro include thiopurine analogs, compound 6 (preclinical), and remdesivir (phase III for MARS-CoV).^121,153^ Favipiravir, ribavirin (randomized trial for SARS-CoV-2), and remdesivir are among the candidate inhibitors of RdRp.^118^ Previously, compounds that may interfere with ATPase and helicase activities were reported (before COVID-19), such as bananins, 5-hydroxychromone derivatives, and triazole derivatives (preclinical).^34,78^ Candidates that may suppress viral entry by targeting the S protein include peptide P9 and α-helical lipopeptides (preclinical),^154^ and those targeting the S2 subunit of the S protein mainly include HR1P, HR1M, HR1L, HR2L, HR2P, and HR2L (preclinical).^155^

Due to the strong affinity between ACE2 receptor and the RBD of SARS-CoV-2, another opportunity might be through the development of antibodies to block ACE2. The second method is to use a large amount of soluble ACE2 to directly block the spike protein. The third method is to find a drug that directly inhibits the spike membrane fusion process, such as the AIDS drug enfuvirtide. The fourth approach is to identify an agent that inhibits the activity of disintegrin and metalloproteinase 17 (ADAM17), which may also prevent viral release and proliferation in cells and protect ACE2 and the lungs.^87^

### mRNA vaccine technology

SARS-CoV-2 vaccines are already under development. The mRNA and DNA vaccines are promising approaches to prevent cellular invasion by similar coronaviruses in the future. In January 2020, the Coalition for Epidemic Preparedness Innovations (CEPI) announced three partnerships to develop a new coronavirus vaccine. Among them, Moderna and Inovio presented mRNA and DNA vaccine technologies, respectively. The goal of the three teams is to have at least one candidate vaccine capable of preventing coronavirus infection in 16 weeks, to be tested in a phase I clinical trial.

Moderna’s vaccine model is designed to use mRNAs to safely pre-expose the immune system to a small amount of encoded proteins usually generated by the pathogen, so that the immune system becomes prepared to fight future pathogens and prevent infection. The candidate genes developed by Moderna contain mRNAs, and combining multiple mRNAs into a single vaccine might be useful to rapidly induce responses, which is an approach that may be applied for future, emerging pandemic threats too. At the same time, because the mRNA in the cell will be degraded over time, the mRNA vaccine will not continue to produce antigen components for a long time, which can avoid the risk of continued stimulation of the immune system. Generally, mRNA vaccines are also described as simple to prepare and to yield remarkable results. Additional advantages, as well as disadvantages, of nucleic acid-based vaccines were described in detail. Furthermore, the basis of traditional vaccines, proteins or polysaccharides, cannot be straight forward applied at present against many pathogens. Therefore, mRNA vaccines would be suitable for achieving rapid, mass production of emergency vaccines in the event of a major epidemic. However, because RNA is easily degraded, inducing cells to absorb mRNA quickly is a challenge. In recent years, the method for delivery of mRNA into cells has been greatly improved, and Stemirna and Moderna have adopted nanolipid (LPP or LNP (lipid nanoparticle)) drug-loading technology. However, this is still to be developed for mRNAs. Furthermore, ensuring safety and effectiveness is another challenge for viral mRNA vaccines, and clinical validation will have to be carried out carefully.

Moderna is a worldwide leader in mRNA therapy, with currently nine mRNA-type vaccine candidates (e.g., mRNA-1273 against SARS-CoV-2). The National Institutes of Allergy and Infectious Diseases (NIAID) of the National Institutes of Health (NIH) and the mRNA vaccine giant Moderna have teamed up to bring the new coronavirus vaccine possibly to phase II within a few months, and phase III late this year. Simultaneously, in January 2020, the Translational Medicine Platform of Dongfang Hospital affiliated with Tongji University cooperated with Stemirna (Shanghai) Biotechnology Co., Ltd. to rapidly promote the development of a new coronavirus mRNA vaccine. In February 2020, the Chinese scientific research team announced that animal testing of the newly developed coronavirus vaccine has begun. If animal testing goes well, this new vaccine will enter human clinical trials within a few months. Also in February 2020, Zhuhai Livanda Biotechnology Co., Ltd. announced that the first batch of new coronavirus mRNA vaccine standard samples completed during the Spring Festival had been delivered to relevant national authorities on for animal testing and efficacy verification. The researchers detected the production of target antibodies in mouse serum at day 12 following immunization. The company claimed that this was also the first time worldwide that new coronavirus vaccines developed based on mRNA technology have resulted in antibodies in animals. Livanda is currently pushing ahead with the project through extensive cooperation with the Academy of Military Medical Sciences, the Guangdong Provincial Institute of Supervision, and the Macau University of Science and Technology. The antigen used in its development is the same as that used by Moderna.

### DNA vaccine technology

DNA vaccine technology may have many advantages (e.g., safe, fast, less technical barriers), along with potential limitations too.^156^ A phase I human clinical trial of the MERS-CoV vaccine has proven its safety and effectiveness.^157^ The DNA vaccine based on the SARS-CoV S protein developed by the team of Barney Graham and Gary Nabel of the US-NIH Vaccine Research Center (VRC) has achieved positive results in animal experiments and a clinical phase I trial.^158^^,159^ Inovio Pharmaceuticals has accumulated extensive safety and immunogenicity data for MERS-CoV vaccine studies. Because of the high degree of similarity between MERS-CoV and SARS-CoV-2, lessons from such a vaccine development process may be beneficial for the development of a DNA vaccine against SARS-CoV-2. Since January 2020, Inovio has been collaborating with several experts and companies (some in China), and has currently already begun phase I clinical trials of the DNA vaccine INO-4800.

### Recombinant vaccines

China’s CanSino Biologics might be the leader as it was announced it has moved to phase II testing of a vaccine called Ad5-nCoV. The latter is currently often being reported as a DNA vaccine, but to be precise, it uses viral vectors (adenovirus) to deliver DNA related to SARS-CoV-2. Part of the project is carried out currently with the Chinese military medical research institute. The vaccine candidate is constructed using genetic engineering methods and defective human type 5 adenovirus as a vector that can express the SARS-CoV-2 S antigen, to stimulate the body to produce strong humoral or cellular immunity.

## Conclusion

SARS-CoV and MERS-CoV belong to subgroups 2b and 2c of the betacoronaviruses, respectively, and SARS-CoV-2 is a new member of betacoronaviruses, distinct from SARS-CoV and MERS-CoV. SARS-CoV and SARS-CoV-2 are similar in terms of invasion and self-replication, whereas MERS-CoV has different targets. The cellular receptor of SARS-CoV and SARS-CoV-2 is ACE2, plus CD147 for SARS-CoV-2, while that of MERS-CoV is DPP4 (CD26). All of them evolved a mechanism to escape the host cell immune system. SARS-CoV and SARS-CoV-2 affect the RAS system by suppressing ACE2, leading to the onset of symptoms. MERS-CoV causes symptoms by producing inflammatory cytokines and invading T cells. SARS-CoV, MERS-CoV, and SARS-CoV-2 infections have similar symptoms, including fever, cough, myalgia, and shortness of breath, among others. Current treatments for SARS mainly include IFN-α, antiviral treatments (e.g., ribavirin), plasma therapy, host-directed therapies, and systemic corticosteroids. For MERS, several therapeutics are in development, including convalescent plasma, lopinavir/ritonavir, ribavirin, IFN, and novel therapies, including polyclonal antibodies, broad-spectrum antivirals, and AMPs. For COVID-19, candidates mainly include drugs targeting the S protein, nonstructural proteins (3CLpro, PLpro, helicase, and RdRp), and viral RNA synthesis blockers such as Remdesivir and Ribavirin. Vaccines such as DNA vaccine, mRNA vaccine, and recombinant vaccines are being rapidly developed.

So far this century, human beings have experienced several epidemic outbreaks, and each outbreak had a negative impact at different levels, including health, economy, and even psychology and human behavior. In the future, more precautious measures should be available to guide individuals and groups to take effective emergency measures and to support social stability, and physical and mental health. Furthermore, additional studies of coronaviruses and disease epidemics should continue, to support the preparation for future responses, medical therapies, vaccines, and methods of relieving personal anxiety. This review has highlighted several approaches, and the names and progress related to several compounds and biologics currently under research and development, as well as the companies and researchers involved in these efforts. For future directions, it has also described the differences and similarities, as well as the potential routes and possibilities for targeting each of the main three viruses investigated here.
